# The role of cDC1s *in vivo*: CD8 T cell priming through cross-presentation

**DOI:** 10.12688/f1000research.9997.1

**Published:** 2017-02-01

**Authors:** Derek Theisen, Kenneth Murphy

**Affiliations:** 1 Department of Pathology and Immunology, Washington University in St. Louis School of Medicine, St. Louis, MO, USA; 2 Howard Hughes Medical Institute, Washington University in St. Louis School of Medicine, St. Louis, MO, USA

**Keywords:** Dendritic cell, monocyte-derived dendritic cells, T cell response

## Abstract

The cDC1 subset of classical dendritic cells is specialized for priming CD8 T cell responses through the process of cross-presentation. The molecular mechanisms of cross-presentation remain incompletely understood because of limited biochemical analysis of rare cDC1 cells, difficulty in their genetic manipulation, and reliance on *in vitro* systems based on monocyte- and bone-marrow-derived dendritic cells. This review will discuss cross-presentation from the perspective of studies with monocyte- or bone-marrow-derived dendritic cells while highlighting the need for future work examining cDC1 cells. We then discuss the role of cDC1s as a cellular platform to combine antigen processing for class I and class II MHC presentation to allow the integration of “help” from CD4 T cells during priming of CD8 T cell responses.

## Introduction

Dendritic cells (DCs) are a distinct lineage of innate immune cells that was originally defined based on its unique stellate morphology and ability to prime T cell responses ^1– 3^. DCs broadly segregate into four groups, plasmacytoid DCs (pDCs), classical DCs (cDCs), Langerhans cells, and monocyte-derived DCs (moDCs) based on function and surface markers. pDCs are potent producers of type I interferons in response to viral pathogens ^4– 7^. cDCs themselves are divided into two lineages, recently renamed ^8^ as cDC1 (CD8α ^+^ DCs) and cDC2 (CD8α ^˗^ DCs). The cDC2 lineage is heterogeneous and expresses the *Irf4* transcription factor ^9– 11^. Notch 2-dependent cDC2s are required for IL-23 production in response to *Citrobacter rodentium* infection ^12, 13^, while a separate *Klf4*-dependent subset of cDC2s is required for type II responses to house dust mite antigen and *Schistosoma mansoni* infection ^14^. By contrast, cDC1 cells require the *Irf8* ^10, 15, 16^ and *Batf3* transcription factors ^10, 16, 17^ and produce the IL-12 necessary for protection against *Toxoplasma gondii* ^18, 19^. They are also the subset involved in priming CD8 T cell responses to tumors and virally infected cells through cross-presentation ^17, 20^. All cDCs *in vivo* arise from a common DC progenitor (CDP) in the bone marrow ^21^.

Cultures of monocytes in GM-CSF and IL-4 are able to produce DC-like cells, distinct from those that develop from the CDP ^22^, termed monocyte-derived DCs (moDCs), in large numbers ^23^. Similar cells that derive from cultures of whole bone marrow with GM-CSF with or without IL-4 *in vitro* have been referred to as “moDCs”, despite the uncertainty of the origin, or bone-marrow-derived DCs (BMDCs). BMDCs have been the basis for many studies aimed at understanding the properties of cDCs ^24, 25^. Recent studies have shown that these cultures are actually heterogeneous and that it may not be appropriate to refer to the cells that are generated as moDCs, since many display macrophage characteristics and the precursor to the DC-like cells from whole bone marrow is not known ^26^. Some investigators object to the use of the term moDC for *in-vitro*-derived cells from whole bone marrow since it is misleading with regard to their development; however, it has been argued that the DC-like cells that develop from GM-CSF cultures develop from monocytes ^27^. The term BMDC can also lead to confusion, since DCs can also be derived from bone marrow cultures with fms-related tyrosine kinase 3 ligand (Flt3L) and produce cells that are distinct from those produced in GM-CSF cultures ^27^. Therefore, in this review, we will refer to cells generated from monocytes as moDCs and cells generated from whole bone marrow GM-CSF cultures as GMDCs. Conceivably, both may be derived from monocytes and distinct from *in-vivo*-derived cDCs.

In this review, we will first highlight new discoveries regarding cross-presentation and discuss how molecular mechanisms governing cross-presentation by cDC1s may be distinct from the cross-presentation pathways identified in moDCs or GMDCs *in vitro*. We will then describe how cDC1s initiate and maintain anti-viral responses, including through their interactions with CD4 T cells.

## Molecular mechanisms of cross-presentation

Cross-presentation is the process by which exogenous antigens are taken up by antigen-presenting cells and presented on major histocompatibility class I (MHCI) ^28^. cDC1s are the unique DC subset specialized in cross-presentation *in vivo* ^20^. The molecular mechanisms specific to DCs that govern cross-presentation have been the subject of a large body of work over the past decade ^29^, with much of the early work on cross-presentation carried out in macrophages ^30– 32^, though the majority of our understanding of cross-presentation is based on experiments carried out using GMDCs. GMDCs are generated from bone marrow cultures with GM-CSF alone or GM-CSF with IL-4 originally developed in the early 1990s ^24– 26^. While these cells can cross-present *in vitro*, it is unlikely that these are the cells that operate *in vivo*, since *Batf3 ^-/-^* mice that lack cDC1s fail to mount CD8 T cell responses to challenges requiring cross-presentation ^17^. However, *Batf3 ^-/-^* mice can generate moDCs that are able to cross-present normally *in vitro* ^33^, indicating that any moDCs that may develop *in vivo* do not compensate for the loss of cDC1s for *in vivo* cross-presentation.

Surprisingly little work has been done to analyze cross-presentation in DCs derived from bone marrow cultures with Flt3L. DCs that resemble splenic cDC1 and cDC2 by surface markers can be generated in large numbers in bone marrow cultures with Flt3L ^34, 35^. These cells are able to present antibody-targeted antigens and activate T cells to a similar extent as cDCs of the same lineage derived *in vivo* ^36^. Also, Flt3L-derived DCs express *Rab43*, a molecule necessary for cross-presentation in *in vivo* cDC1s but not moDCs ^37^. While more studies may be needed to compare the cross-presentation efficiency of Flt3L-derived DCs to *in-vivo*-generated cDCs, Flt3L-derived DCs are arguably more appropriate for *in vitro* studies of DC function than GMDCs. Nonetheless, the examination of macrophages and GMDCs has been useful for identifying the components of two major cross-presentation pathways, the cytosolic and vacuolar pathways.

In the cytosolic pathway, exogenous antigens that are taken up into phagosomes are exported into the cytosol to enter the traditional proteasome- and TAP-dependent MHCI presentation pathway ^32, 38, 39^. The cytosolic pathway is dependent on the reduced acidification of phagosomes produced by the activity of NADPH oxidase Nox2, leading to delayed antigen degradation ^40, 41^. Recruitment and localization of NOX2 components was determined to be regulated by the activities of Rac2 and Rab27a ^41, 42^. Phagosomal alkalization has also been demonstrated to involve Rab3c (a marker of recycling vesicles ^43^), Rab34 (an LPS-regulated protein that can delay phago-lysosomal fusion ^44^), and TFEB (a transcription factor that can negatively regulate cross-presentation ^45^). The delay in antigen degradation caused by phagosomal alkalization acts to allow antigens to move into the cytosol, possibly through channels such as Sec61, promoting antigen processing and presentation through the normal MHCI pathway ^46^. These pathways have mainly been shown to act in phagosomes containing latex beads, raising the question of whether this process is specific to uptake of beads or if antigens that bind different receptors are processed through similar mechanisms.

NOX2 has been shown to play a role in cross-presentation *in vivo* ^40, 42^, suggesting that phagosomal alkalization may also be important for cross-presentation by cDC1s. However, the magnitude of the contribution of this pathway is limited, as loss of NOX2 activity decreased cross-presentation of antibody-targeted antigen only by about 50% ^40^. The remainder of the molecules in the cytosolic pathway, including Rac2, Rab27a, Rab3c, Sec61, TFEB, and Rab34, have not been examined in *in vivo* cDCs ^41– 45^. Genetic studies with mouse models will be necessary to determine the importance of these molecules and the cytosolic pathway in general to cross-presentation *in vivo*.

The vacuolar pathway involves the loading of MHCI molecules by antigens processed directly within endosomes without transport to the cytosol and is independent of TAP and the proteasome ^47, 48^. One molecule linked to the vacuolar pathway is the insulin-regulated aminopeptidase (IRAP) ^49^. IRAP can trim peptides in DC phagosomes to lengths appropriate for loading into MHCI molecules ^49^, similar to the action of endoplasmic reticulum aminopeptidase associated with antigen processing (ERAAP) in the endoplasmic reticulum ^50^. The role of IRAP *in vivo* remains unclear. Although an early study detailing the mechanism of IRAP was conducted using *in vitro* GMDCs, IRAP-deficient mice were also shown to have reduced cross-presentation ^49^. However, a subsequent study concluded that IRAP was not required for cross-presentation of soluble OVA or OVA-coated splenocytes by splenic cDC1s *in vitro*, suggesting that IRAP may not play a role in cDC1-mediated cross-presentation ^51^. But another study, using OVA-expressing yeast *in vitro*, showed that IRAP is recruited to endosomes in cDC1 cells and that cross-presentation is reduced in IRAP-deficient cDC1s ^52^. Conceivably, the use of differing forms of antigen underlies some of these variances. While the ability of cDC1 cells to cross-present is not solely due to their ability to capture antigens ^53, 54^, it is plausible that distinct antigen internalization and processing pathways are used for different forms of antigen. For example, cell-associated and soluble antigens are not cross-presented equally and cDC2s, which do not cross-present *in vivo* ^20^, have the capacity to present soluble antigens *in vitro* ^52, 54^. Therefore, work is still needed to compare cross-presentation of different antigens by cDC1s and cDC2s *in vitro* to find a system that mimics *in vivo* models where only cDC1s are able to cross-present. Developing standardized assays for the field through careful comparison of DC subsets may help to eliminate confusion between whether or not molecules are necessary for cross-presentation *in vivo* as in the case of IRAP.

Presentation through the vacuolar pathway requires the loading of MHCI molecules within endosomes. The molecule Sec22b was described in GMDCs to regulate the movement of the peptide-loading complex to endosomes ^55^. It has also been shown that GMDCs contain pools of MHCI in endosomal recycling compartments marked by Rab11a ^56^. A model has been proposed where TLR signals induce MHCI movement from these intracellular pools to phagosomes, where they meet antigen and the peptide-loading complex machinery brought by Sec22b ^56^. A second proposed model involves CD74, the MHCII invariant chain, which was also shown to control the movement of MHCI to endosomes and to regulate cross-presentation *in vivo* ^57^. CD74 acts in both splenic cDC1s and GMDCs, meaning CD74 and IRAP are the two molecules shown to be involved in the vacuolar pathway of cross presentation in cDC1s ^52, 57^. However, as with the cytosolic pathway, many gaps still remain in our understanding of what proteins and signals are involved in regulating the cross-presentation ability of cDC1s.

## Role of moDCs *in vivo*

The discovery that moDCs cannot compensate for the loss of cross-presentation by cDC1s *in vivo* has called into question their relevance *in vivo* ^33^. Bone marrow cultured with GM-CSF produces a heterogeneous population of CD11c ^+^ MHCII ^+^ cells which contain functionally distinct macrophages and DCs ^26^. While moDCs have a stellate morphology, express the cDC-specific ZBTB46 transcription factor ^58^, and can cross-present cell-associated antigens, they do so in a manner distinct from *ex vivo* cDC1 cells ^33, 51^.

Further, recent work has called into question if moDCs exist *in vivo*. Studies of moDCs started with the observation that transferred monocytes are able to generate CD11c ^+^ DC-like cells *in vivo* ^22^. These moDCs have been observed in numerous models including viral infections ^59^, alum-OVA immunization ^60^, arthritis ^61^, and house dust mite exposure ^62^. They can be distinguished from cDCs *in vivo* by expression of CD64 and MAR-1 ^60, 62^ and are dependent on CCR2 and CD115 (MCSF-R) ^60, 63^. However, it is unclear whether the moDCs identified in these studies *in vivo* are equivalent to those generated with GM-CSF and IL-4 *in vitro*. Recent lineage tracing has suggested that the inflammatory cells that develop during house dust mite challenge lack expression of the cDC marker ZBTB46 ^58^ and instead express the macrophage-specific transcription factor MafB ^64, 65^, suggesting that these cells are not moDCs but rather monocyte-derived macrophages. Furthermore, others have shown little functional difference among moDCs, monocyte-derived macrophages, myeloid-derived suppressor cells, and immature monocytes ^8, 66, 67^, also suggesting that *in vivo* moDCs may actually be monocyte-derived macrophages. In addition, no *in vivo* model has yet to be described where moDCs are required for cross-presentation. Lineage tracing of *in vivo* moDCs and comparisons to *in vitro*-derived GMDCs will be necessary to determine whether GM-CSF cultures are an appropriate model to study DC function. Owing to the observed differences between GMDCs and cDC1s, studies of cross-presentation *in vitro* should rely on either *ex vivo* cDCs or Flt3L-derived DCs to more appropriately model how cross-presentation occurs *in vivo*.

## Cross-presentation during viral infections

**Figure 1.**
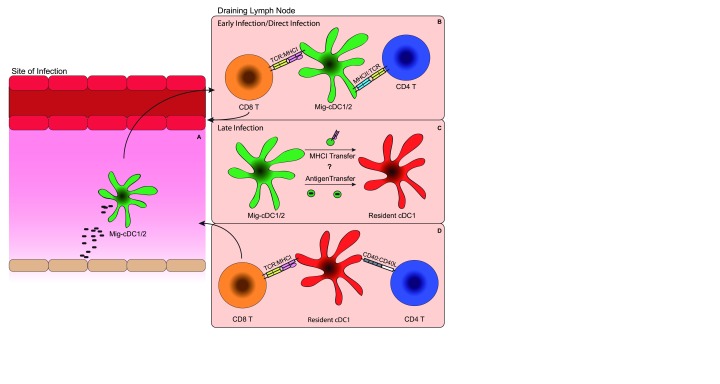
Model for CD8 T cell priming by resident classical CD8α ^+^ dendritic cells (cDC1s). ( **A**) Antigen is captured by migratory cDC1s or CD11b ^+^ cDCs (cDC2s) at the site of infection by either direct infection or phagocytosis. ( **B**) After antigen capture, migratory cDC1s or cDC2s with antigen then migrate to the draining lymph node, where they prime naïve antigen-specific CD4 and possibly CD8 T cells through major histocompatibility (MHC):T cell receptor (TCR) interactions. ( **C**) Migratory cDCs transfer antigens to resident cDC1s through either “cross-dressing”, the process by which loaded MHCI is transferred between cell membranes, or by transferring the antigen itself, which is then taken up by the resident cDC1s for cross-presentation. ( **D**) Resident cDC1s receive “help” through CD40:CD40L interactions with CD4 T cells, which allow them to prime antigen-specific naïve CD8 T cells through MHCI:TCR interactions. Mig-DC, migratory dendritic cell.

Though cDC1s are the major cell that appears to carry out cross-presentation for expanding CD8 T cells *in vivo* ^20^, many cells are able to present antigens on MHCI to CD8 T cells ^68^. Therefore, it is unclear whether cross-presentation is the only pathway used in priming CD8 T cells to pathogens, or alternately whether direct presentation by infected cells might contribute in some settings. Indeed, the cell type responsible for T cell priming and the pathway of antigen processing may vary with the pathogen and could depend on factors such as viral tropism and the time after infection ^69– 71^. For example, using DC-tropic vaccinia virus expressing an extended OVA peptide that could not be cross-presented, Xu *et al*. demonstrated that direct presentation is sufficient for generating a CD8 T cell response ^69^. However, during infection with mouse cytomegalovirus, another DC-tropic virus, the predominant T cell clones react to epitopes that were presented through cross-presentation ^70, 71^. It is likely that both direct and cross-presentation can contribute in priming CD8 T cell responses and that the predominant form of presentation may depend on the stage of infection. Early during infection, antigen presentation requires viral replication, suggesting direct presentation is playing a role; however, late during infection most presentation occurs by uninfected DCs through cross-presentation ^72^ ( Figure 1). Imaging of T cells and cDC1s during vaccinia virus infection showed a similar phenomenon and it was observed that multiple DC subsets could prime CD8 T cell responses early during infection; however, later in infection CD8 T cells interacted with only XCR1-expressing cDC1s ^73^. cDC1s are also essential for priming CD8 T cell responses during secondary infections and generating T resident memory cells, a process recently shown to depend on cross-presentation ^74, 75^.

In lymph nodes, cDC1s can be separated into two categories of migratory and resident DCs that are developmentally related ^76^, either of which could be involved in the presentation of antigen to CD8 T cells during an infection. Tracking of migratory DCs from the skin during herpes simplex virus (HSV) infection has shown that CD8 T cell priming occurs in lymph nodes and movement of migratory DCs from the skin is required for priming to occur ^77^. Then in the lymph node, antigens acquired by migratory DCs can be transferred to lymph-node-resident DCs for presentation to CD8 T cells ^77^ ( Figure 1C). These results imply that there may be two distinct priming events: an initial priming from migratory cDC1s that directly captured antigen and then a secondary priming that occurs after antigen has been transferred to resident cDC1s. Imaging of the anti-viral response to HSV suggested that CD4 T cells are primed before CD8 T cells and that they interact with migratory DCs, while CD8 T cells interact with resident cDC1s in the lymph node ^78^. However, others have demonstrated that antigen-specific CD8 T cells preferentially interact with migratory cDC1s ^79, 80^. These results raise the question of whether all CD8 T cell priming occurs through migratory cDC1s, which are directly exposed to antigens, or through resident cDC1s, which can present their antigens through either cross-presentation ^73, 77^ or cross-dressing, a process by which loaded MHCI is transferred between different cells ^81^. Conceivably, early CD8 and potentially CD4 T cell priming is mediated by direct presentation from migratory cDC1s, since they encounter antigen first, and then later CD8 T cell priming occurs after antigen transfer to and cross-presentation by lymph-node-resident cDC1s ( Figure 1A and D).

## CD4 T cells and cDC1s

For many pathogens, DCs alone are not enough to prime a CD8 T cell response. CD4 T cells and type I interferons have been shown to be involved in the “help” reaction, which stimulates DCs and enables them to prime CD8 T cells ^82, 83^. Early work on cross-presentation showed that CD4 T cell help to DCs is necessary for the generation of a CD8 T cell response against cell-associated antigens ^82^. This help is mediated through interactions between CD40 on DCs and CD40L on CD4 T cells ^84– 86^. These results describe a “bridge” model, where CD4 T cells and CD8 T cells interact with the same dendritic cell, albeit likely at different times, in order to properly prime a cytotoxic T cell response ^78, 85^. This suggests that CD4 T cells must be activated prior to CD8 T cells, likely by migratory cDCs, in order for them to act on cDC1s through CD40L to help induce CD8 T cell priming ( Figure 1B and D).

Questions remain as to whether the interaction between cDC1s and CD4 T cells is antigen specific. Initial studies that showed that CD4 T cell help for CD8 T cell priming required cognate CD4 T cell interactions ^82^. However, later it was suggested that CD40 signaling was sufficient to provide help, even when DCs lack MHCII ^85^. *In vitro* analysis of presentation by DC subsets using antibody-targeted antigen implied that cDC1s were relatively poor in antigen presentation to CD4 T cells relative to cDC2s, while cDC2s were adept at activating CD4 T cells *in vitro* ^36, 87^. This leads to the question of whether cDC1s use MHCII presentation solely to obtain help from previously activated CD4 T cells for CD8 T cell priming or, alternatively, whether cDC1s can also prime naïve CD4 T cells. A recent study has shown that CD8 T cells cluster with cDC1s, while CD4 T cells cluster with cDC2s during OVA immunization ^88^, suggesting that T cell priming may be DC-subset specific. However, late during viral infection both cDC1 and cDC2 subsets have the capacity to activate CD4 T cells ^79^. In addition, both CD4 and CD8 T cell priming against insulin in non-obese diabetic mice is decreased in the absence of cDC1s ^89^. Since CD4 T cells were shown to be primed first by migratory DCs ^78^, it is possible that migratory cDC1s prime the CD4 T cells that later help lymph-node-resident cDC1s induce CD8 T cell priming ( Figure 1B and D). Further studies will be necessary to determine to what extent each DC subset contributes to T cell priming in different infection contexts.

## Conclusion

cDC1s are the predominant cross-presenting cells functioning in CD8 T cell priming *in vivo* ^20^. Recent imaging studies suggest that cDC1s also function as a platform for CD4 T cell help during viral infections ^74, 78^, likely through CD40–CD40L interactions ^84, 85^. However, it remains unclear whether cDC1s can also prime naive CD4 T cells or whether they receive only help from them ^36, 79, 82, 89^. More sophisticated *in vivo* models will need to be generated in order to determine the role of cDC1s in priming CD4 T cell responses *in vivo* in order to further distinguish the unique roles of DC subsets.

Transcriptional profiling has suggested that moDCs may not be a functional cross-presenting DC subset *in vivo* ^33^ and at least in one setting may represent monocyte-derived macrophages ^65^. Many molecules described previously to be involved in cross-presentation were evaluated in the context of GMDCs and need to be examined in the context of cDC1s ^41– 45^. Recent advances in DC biology have allowed for the conditional deletion of genes in cDC1s through the use of XCR1-cre ^90^ and analysis of transcriptional differences between DC subsets ^91^. Examining molecules described in moDCs also in cDC1s and studying other cDC1-specific genes will aid in our understanding of how cross-presentation against viral and cancer antigens occurs and may provide more insight into whether moDCs are a true DC subset *in vivo*. Elucidating the mechanisms by which cDC1s activate CD8 T cells and the mechanisms underlying the various interactions between DC subsets and T cells should be of value in designing DC-based cancer vaccines.

## Abbreviations

BMDC, bone-marrow-derived dendritic cell; cDC, classical dendritic cell; CDP, common dendritic cell progenitor; DC, dendritic cell; Flt3L, fms-related tyrosine kinase 3 ligand; GMDC, GM-CSF-derived dendritic cell; HSV, herpes simplex virus; IRAP, insulin-regulated aminopeptidase; MHCI, major histocompatibility class I; MHCII, major histocompatibility class II; moDC, monocyte-derived dendritic cell; pDC, plasmacytoid dendritic cell.
